# Particle-Stabilized Fluid-Fluid Interfaces: The Impact of Core Composition on Interfacial Structure

**DOI:** 10.3389/fchem.2018.00383

**Published:** 2018-08-30

**Authors:** Alison Tasker, Frank Sainsbury, Simon Puttick

**Affiliations:** ^1^Australian Institute for Bioengineering and Nanotechnology, University of Queensland, Brisbane, QLD, Australia; ^2^Commonwealth Scientific and Industrial Research Organisation, Probing Biosystems Future Science Platform, Brisbane, QLD, Australia

**Keywords:** VLPs, pickering emulsions, polymer microcapsules, interfacial chemistry, drug delivery

## Abstract

The encapsulation of small molecule drugs in nanomaterials has become an increasingly popular approach to the delivery of therapeutics. The use of emulsions as templates for the synthesis of drug impregnated nanomaterials is an exciting area of research, and a great deal of progress has been made in understanding the interfacial chemistry that is critical to controlling the physicochemical properties of both the encapsulated material and the templated material. For example, control of the interfacial tension between an oil and aqueous phase is a fundamental concern when designing drug delivery vehicles that are stabilized by particulate surfactants at the fluid interface. Particles in general are capable of self-assembly at a fluid interface, with a preference for one or the other of the phases, and much work has focussed on modification of the particle properties to optimize formation and stability of the emulsion. An issue arises however when a model, single oil system is translated into more complex, real-world scenarios, which are often multi-component, with the incorporation of charged active ingredients and other excipients. The result is potentially a huge change in the properties of the dispersed phase which can lead to a failure in the capability of particles to continue to stabilize the interface. In this mini-review, we will focus on two encapsulation strategies based on the selective deposition of particles or proteins on a fluid-fluid interface: virus-like particles and polymer microcapsules formed from particle-stabilized emulsion templates. The similarity between these colloidal systems lies in the fact that particulate entities are used to stabilize fluid cores. We will focus on those studies that have described the effect of subtle changes in core composition on the self-assembly of particles at the fluid-fluid interface and how this influences the resulting capsule structure.

## Introduction

Encapsulation in nanomaterials is a powerful approach to the delivery of active components that require protection from harsh external environments. Encapsulation strategies range from mimicking natural delivery vehicles using virus coat protein self-assembly, through to the stabilization of hydrophobic cores by inorganic nanoparticles or polymeric surfactants to form microcapsules. In the case of delivery vehicles based on a capsule structure, significant effort is directed to optimizing the interface used to template the capsule material, be it polymeric or protein in nature. Based on observations in our laboratory and others (Manuela et al., 2017), the physicochemical properties of the active component itself can have a profound effect on the stability of this interface and this has prompted us to review existing literature in this area.

Whilst it is well-known that the contact angle is a key parameter for stabilization of emulsions using particles (Binks et al., 2007), the literature surrounding the effect of core composition on the organization of stabilizers at the interface is scarce. Thus, in this mini-review, we aim to capture emerging knowledge around the impact of core compositions on interfacial architectures and particle morphology. As compositions are developed with increasing complexity, for example by drug loading, there is an increasing need for fundamental understanding on the assembly of nanoscale to microscale capsules. We aim to bring together information of core effects intended to highlight the importance of consideration of the core properties in particle-stabilized fluid-fluid systems. Herein we consider the selective deposition of both proteins and particles on a fluid-fluid interface: virus-like particles and particle-stabilized emulsion templates. In virus-derived particles, the influence of the core on particle structure can often be mapped to specific changes in the interactions between coat protein subunits. However, for polymer microcapsules, how the changing interactions between the core and particles at the interface affects interfacial organization is somewhat less clear. Here we will focus on capsule-like structures and not solid microparticles where the active ingredient is absorbed into a solid matrix, or covalently bonded to a micro/nanostructure or protein, and the interested reader is instead directed to several recent reviews in these areas (Duncan, 2011; Kopecek, 2013; Chudasama et al., 2016; Han et al., 2016; Ramazani et al., 2016).

## Viruses and virus-like particles

Nature's prototypical delivery vehicles, viruses, are assembled from particulate protein subunits around a nucleic acid or nucleoprotein core. The structural fidelity of capsid subunits enables the rational genetic or chemical modification of solvent-exposed amino acid side chains, which has led to their application in biomedical nanotechnology (Wen and Steinmetz, 2016). The packaging density of viral genomes is such that it is considered to be in a liquid crystalline state (Speir and Johnson, 2012) and the net charge of the interior face of capsid protein subunits is commonly highly positive, imparting a strong preference to assemble around this polyanionic fluid-like core. This feature of capsid subunits has been used to drive the encapsidation of cargo proteins (Glasgow et al., 2012; Brasch et al., 2017) into particles that may be subsequently surface-modified for cellular uptake (Ashley et al., 2011). As spherical virus capsids can undergo elastic deformation (Marchetti et al., 2016), it is perhaps not surprising that there is some plasticity in the interfacial arrangement of coat protein subunits around various core compositions. For example, the packaging of DNA-tagged enzymes into Cowpea chlorotic mottle virus (CCMV) capsids results in a radial swelling that changes the way capsid subunits interact with each other at the interface, although the particle remains stable (Brasch et al., 2017).

**Figure 1:**
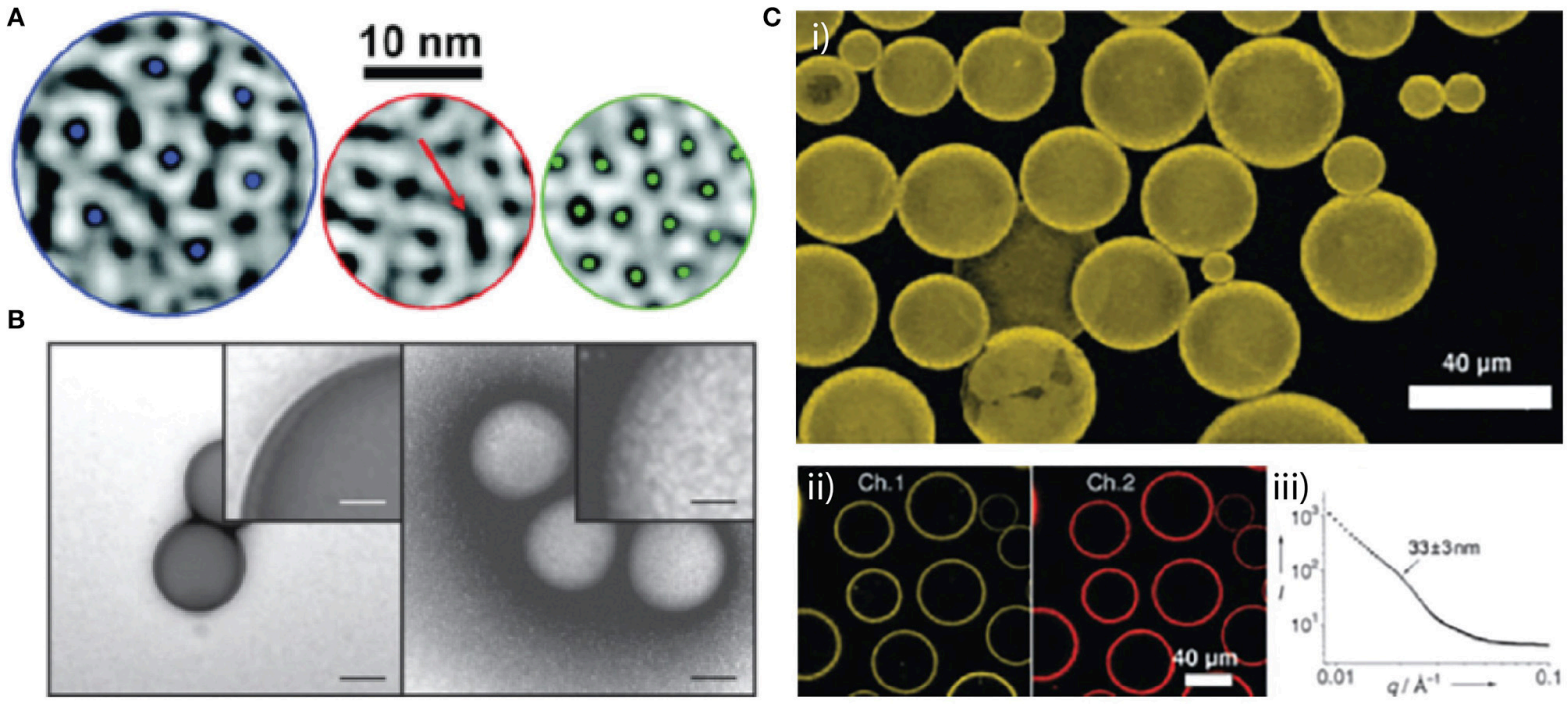
Examples of virus interfacial arrangements around various core compositions. **(A)** Local CCMV protein structures observed on the surfaces of nanodroplets have different degrees of order and disorder. **(A)** Left: six-fold-coordinated capsomers (blue dots at center) represent a high degree of order seen mostly on smaller droplets. Middle: an example of a trough-like scar that consists of an elongated dark region (red arrow) surrounded by a protruding white region. Right: hexagonal web structure, typically seen on larger droplets, consisting of dark spots (green dots) surrounded by an interconnected white network of protein protruding from the interface. Reprinted with permission from Chang et al. (2008). **(B)** Polystyrene beads incubated with (right) or without (left) SV40 VP1 pentamers. The samples were visualized by TEM with negative staining. Scale bar = 100 and 25 nm (insets). Reprinted with permission from Kawano et al. (2015). **(C)** (i) A 3D reconstruction of a confocal fluorescence microscope image of perfluorodecalin droplets coated with CPMV/biotin after being cross-linked with avidin for 3 h at 48°C. (ii) A two-channel confocal fluorescence microscope image of particle assemblies after cross-linking with fluorescently tagged streptavidin: Channel 1 shows the CPMV/biotin fluorescence and channel 2 shows the ATTO-655-streptavidin fluorescence. (iii) SAXS data of the cross-linked CPMV/biotin shell around the perfluodecalin droplets. Reprinted with permission from Russell et al. (2005).

While capsid morphologies are constrained by a limited range of subunit interactions, these interactions can be altered by the encapsidated cargo. For example, Hu et al. demonstrated that the capsid subunits of CCMV assembled around an anionic polymer, poly(styrene-sulfonate) can be forced into assemblies of 120 or 180 subunits depending on the molecular weight of the polymer (Hu et al., 2008). Capsids of the related Brome mosaic virus (BMV) assembled around different segments of the BMV genome, while all the same size and composed of 180 capsid subunits, display different physical properties that impact the rate of interfacial disassembly and cargo release (Vaughan et al., 2014). In these examples, regular subunit lattices are maintained by the conformational switching of capsid protein subunits that allows them to occupy non-equivalent positions on the native icosahedron. Conversely, CCMV subunits deposited on poly(dimethylsiloxane) cores, stabilized by anionic sodium dodecyl sulfate to impart negative charge, forces the subunits into larger and non-icosahedral particles as well as multi-shell structures (Chang et al., 2008). Areas of local subunit organization reminiscent of the native capsid were observed, however, the presence of “scars” indicated core induced disorder at a smaller scale than the subunit (Figure 1A).

The classically pleomorphic members of the Polyomaviridae have been subject to numerous studies on the impact of core composition on capsid geometry. For example, varying the ratio of linear DNA to capsid subunit of SV40 can be used to control size, shape, and stability of virus-like particles (Mukherjee et al., 2010). unrivaled flexibility in the lateral interactions between polyomavirus capsid subunits allows them to assemble on cores considerably larger or smaller than the native 50 nm capsid, while maintaining a regular subunit lattice, for example polystyrene beads of 100 and 200 nm (Figure 1B; (Kawano et al., 2015)), or the much smaller 8, 20, and 27 nm citrate-coated oleate-iron nanoparticles (Enomoto et al., 2013).

Whole virus capsids have also been used to stabilize fluid-fluid interfaces. Their amenability to genetic or chemical modification makes them particularly attractive for the formation of hierarchically ordered structures based on Pickering emulsions. In an early example, Russell et al. showed that chemically labeled Cowpea mosaic virus (CPMV) particles assembled as a monolayer at the interface of perfluorodecalin emulsions (Figure 1C; (Russell et al., 2005)). Chemical crosslinking of unmodified particles, or biochemically linking biotinylated particles with streptavidin, resulted in non-equilibrium stability of the capsules and the dual fluorescence-biotin labeling demonstrated the excellent potential for further functionalization. Turnip yellow mosaic virus (TYMV) was similarly used to stabilize a perfluorodecalin emulsion with fluorescent functionalization of the TYMV particles (Kaur et al., 2009). Kaur et al. report that among six different core compositions stable emulsions could only be formed with perfluorodecalin, highlighting the importance of particle-core compatibility in the formation of such capsules (Kaur et al., 2009). Furthermore, this study showed that under the dynamic conditions of emulsification the monolayer of particles is disorganized, despite the ability of the icosahedral virus capsid to form ordered hexagonal arrays. While both CPMV and TYMV are spherical viruses with icosahedral symmetry, Tobacco mosaic virus (TMV) is helical with a rod-shaped morphology. In a further example, He et al. showed that the anisotropic morphology of TMV could be exploited to generate dramatically different interfacial organization of the particle. At low particle concentrations rod-shaped particles were oriented parallel to the interface of a perfluorodecalin core, however, at high concentrations segregation at the interface forced the rods to orient perpendicular to the interface, overcoming inter-particle electrostatic repulsion (He et al., 2009).

## Polymer microcapsules templated on a particle-stabilized fluid-fluid interface

The use of a fluid-fluid interface as a template for the synthesis of polymer microcapsules encapsulating an active component is an exciting area and a great deal of progress has been made in understanding the interfacial chemistry that is critical to controlling the physicochemical properties of both the encapsulated material and the templated material. Control of the interfacial tension between the three phases in an emulsion system (core, aqueous, and stabilizer) is a fundamental concern when designing a template emulsion and one approach is to use solid nanoparticles as the stabilizer to form a Pickering emulsion. Particles in general are capable of self-assembly at a fluid interface, for example in an oil-water (o/w) emulsion, with a preference for one or the other of the phases and much work has focussed on tailoring the surface chemistry of the particle to control properties such as surface activity and to optimize emulsion properties such as stability and cargo release (Aveyard et al., 2003; Amalvy et al., 2004; Read et al., 2004; Ngai et al., 2006; Tasker et al., 2018). An issue arises however when a model, single oil system is translated into more complex, real-world scenarios, which are often multi-component, with the incorporation of charged active ingredients and other excipients. This potentially alters the properties of the dispersed phase leading to changes in the particle contact angle, which may result in a reduced capacity of particles to stabilize the interface.

**Figure 2:**
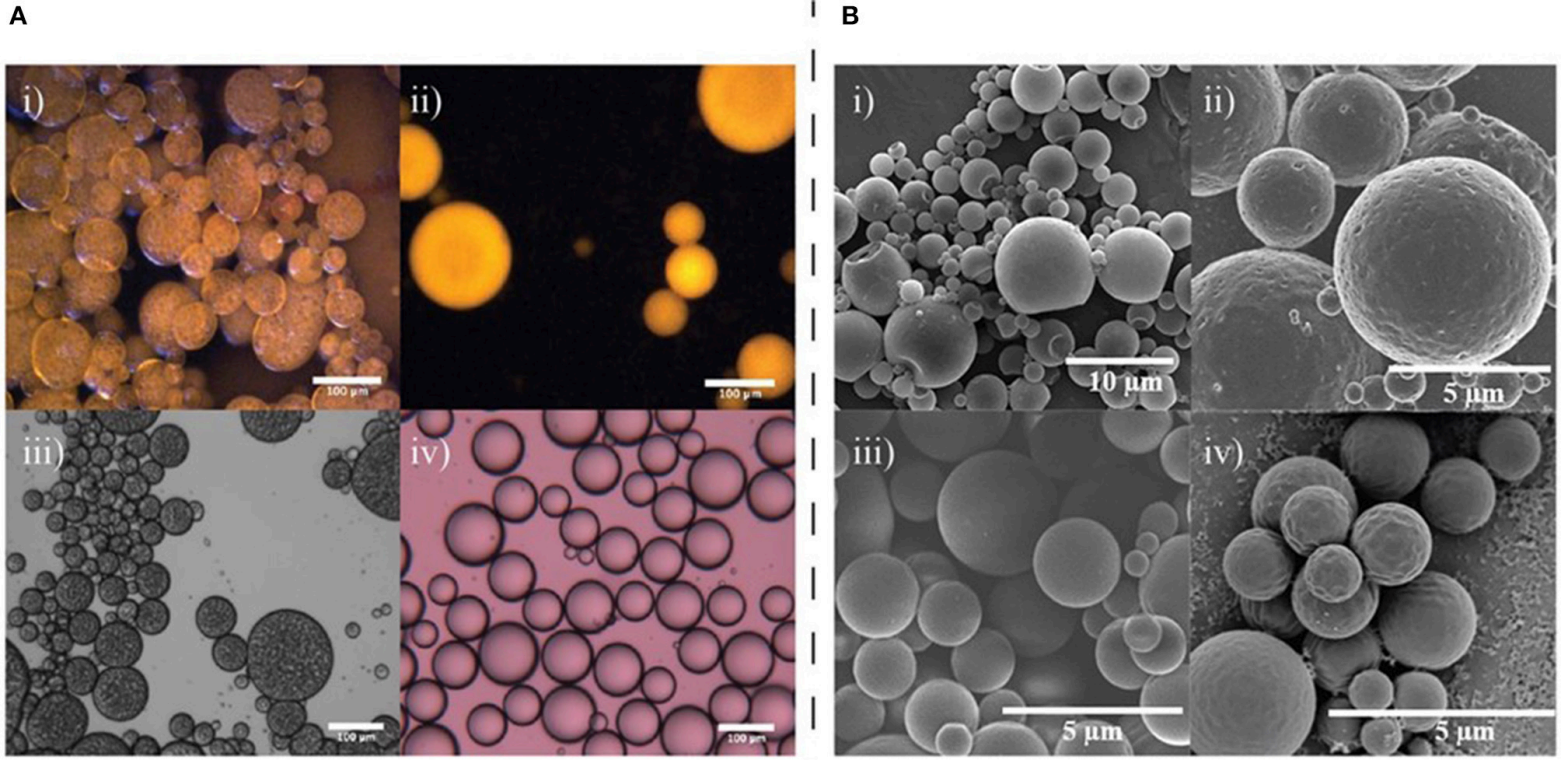
Examples of how core composition can affect the structure of fluid-fluid interfaces and resultant templated microcapsules. **(A)** Fluorescence and transmission optical micrographs showing multiple emulsion formation for olive oil and single emulsion formation for toluene with aqueous GO solutions; (i) fluorescence image of an olive oil emulsion, (ii) fluorescence image of a toluene emulsion, (iii) optical microscopy image of an olive oil emulsion, (iv) optical microscopy image of a toluene emulsion. All samples were prepared with 1 mg/mL GO at pH 1. Reprinted with permission from Ali et al. (2017). **(B)** The changing morphology of poly(methyl methacrylate) microcapsules when the core composition is modified between (i) Hexadecane, (ii) hexyl salicylate, (iii) cyclamen aldehyde, and (iv) toluene. Reprinted with permission from Tasker et al. (2016).

For example, graphene oxide (GO) sheets have been successfully used to stabilize emulsions with either a water-oil-water (w/o/w) or oil-water (o/w) morphology, depending on the oil used as the dispersed phase, via a Pickering emulsion formation (Ali et al., 2017). The authors found that for both toluene and olive oil dispersed phases, when the GO concentration was increased, the formed droplets became smaller, suggesting that GO was acting as a limiting interfacial stabilizer. Toluene emulsions required more energy than the olive oil emulsions due to the higher interfacial tension of toluene/water compared to olive oil/water. They also discovered that when olive oil was used as the dispersed phase, multiple emulsions were formed spontaneously, a phenomenon which was not observed with toluene as the dispersed phase (Figure 2A). They suggest that this unusual multiple emulsion formation is due to the more complex mixture of components in the olive oil. Free fatty acids present in the oil contributed to the stability of the internalized water droplets in the w/o/w emulsions that were formed. Furthermore, with toluene, droplet size increased with pH while droplet stability decreased, resulting in mostly coalescence at pH 11, however when olive oil was used, the opposite was seen to be the case—droplets became smaller and more stable with an increase in pH. A minor component of olive oil, oleic acid, is deprotonated at pH 11 to form sodium oleate which is an effective emulsifier and acts to stabilize the emulsions at higher pH. This work highlights the potential for minor components of the core material to play a critical role in the formation and stability of the final emulsion.

Work conducted by Tasker et al. also indicates that the relationship between the interfacial tensions of the three phases involved in polymer microcapsule synthesis, namely the oil, polymer, and aqueous phase, are crucial in determining the final microcapsule morphology as they determine the wettability of the core oil by the polymer in the aqueous phase (Tasker et al., 2016). Although a surfactant is not considered a particle in the traditional sense, this work demonstrates how changing interfacial properties resulting from substitution of the core phase can impact the formation of an emulsion-based polymer microcapsule template. In their study the authors used poly(methyl methacrylate) as the shell-forming polymer using the solvent evaporation method of microcapsule formation with a range of oils and aqueous phases to understand the importance of the correct interfacial behavior. The authors found that when cetyltrimethylammonium bromide was used as the stabilizer and hexadecane was used as the oil phase, acorn morphology microcapsules were formed, whereas when hexadecane was substituted for toluene, cyclamen aldehyde, dihydromyrcenol, or hexyl salicylate, core-shell microcapsules were produced (Figure 2B). This is likely an effect of the hydrophobicity of the oils chosen, as the partition coefficient of hexadecane is much higher than that of the other oils tested. The work demonstrated that interfacial tension and contact angle measurements can help predict whether a given oil-polymer surfactant combination chosen to create a polymer microcapsule will result in desired morphologies. Polarity of the oil phase is a further physicochemical property that has been considered in depth when considering the formation of Pickering emulsions. The polarity of the oil phase can determine what type of emulsion is formed, if any, which again highlights the importance of considering interfacial tension and contact angle measurements in the design of any complex emulsion situation (Binks and Clint, 2002; Read et al., 2004).

The effect of core composition on emulsion stability is also evident when using complex cores with melting points near room temperature. Veverka et al. (2018) made oil-in-gel emulsions using β-glucan as the aqueous phase with a range of natural oils. They found that using conjugated linoleic acid (CLA) as the oil phase in a 1:1 molar ratio with the aqueous phase, using no additional stabilizer, yielded an emulsion which was fully phase separated within 48 h. In comparison, when using cocoa butter as the oil phase, again in a 1:1 ratio, a stable emulsion (up to 12 months) was produced. The authors state that this stability is due to the nature of the cocoa butter, as it contained crystalline particles of saturated fatty acids, as demonstrated by cryo-SEM, and saturated fatty acids are known to behave as solids at the oil-gel interface (Macierzanka et al., 2009; Frasch-Melnik et al., 2010; Ghosh and Rousseau, 2011). This work demonstrates how the core composition is an important parameter to consider when forming an emulsion, as in this case, the presence of particles within the oil actually allowed the unintentional formation of a Pickering emulsion. It is worth noting that when β-glucan particles were added to the gel phase, CLA emulsions were stabilized however no differences in oil droplet properties were observed.

The chain length of core oils can also affect microcapsule morphologies. For example, Wagdare et al. investigated how the use of different oils as the core of Eudragit microcapsules impacted the morphology of the capsule shell (Wagdare et al., 2011). The authors used long chain triglycerides, such as olive oil, coconut oil, and vegetable oil, and the medium chain triglyceride Miglyol, in addition to jojoba oil which is a mixture of monoesters. They found that for the long chain triglycerides, a single core-shell morphology was obtained, but when the medium chain triglyceride was used, multi-compartment core-shell morphologies were obtained. They explain the differences in behavior between the long- and medium-chain triglyceride core oils to be due to their compatibility with the shell material. As the solvent diffuses out of the formed droplets, the concentrations of both the polymer and the oil in the droplet increase. There comes a point at which the polymer is no longer soluble in the remaining solvent and so precipitation/gelation occurs. If the oil has already phase separated before the polymer solidifies, the oil can diffuse through the polymer matrix to form a large droplet in the middle of the capsule. However, if the oil does not phase separate before precipitation/gelation of the polymer, small pockets of oil will become trapped throughout the polymer matrix, resulting in the observed multi-component capsules. Higher molecular weight molecules are less soluble in general than their smaller counterparts and so phase separation will occur at an earlier stage in the capsule formation which explains the difference in morphologies seen for capsules formed using the different oils in this study.

In other cases it has been found that the ratio of components that make up a microcapsule core can also affect the resulting capsule morphology. Wang et al. prepared microcapsules using particle-stabilized emulsion polymerization for applications such as the selective adsorption of bisphenol A (BPA), an endocrine-disrupting chemical found in a wide range of food and drink products (Wang et al., 2018). They first formed an o/w Pickering emulsion, stabilized with silica nanoparticles, with a complex core containing the monomer, 4-vinylpyridine; crosslinker, divinylbenzene; initiator, azobisisobutyronitrile (AIBN); solvent, hexadecane, and the template molecule, BPA. They found that the resulting morphology of the formed polymer capsules could be controlled by adjusting the composition of the oil phase, resulting in either a single void inside the polymer shell, or a multi-component core. Firstly, they note that in the absence of BPA, a single void structure is formed, and this is attributed to the presence of hexadecane, which acts as a non-solvent for the formed polymer, driving it to the oil-water interface where it forms a shell. However, when BPA is added to the oil phase, the internal structure of the capsule changes to what appears to be a series of sub capsules within the external shell. They found that by increasing the BPA concentration in the oil phase, the number of internal cores increases but the cores also decrease in size and, as expected, the shell thickness of the whole capsule decreases as less polymer migrates to the interface. BPA will dissolve in the oil phase if it contains hexadecane at < 50% volume fraction, however, as polymerization occurs the volume fraction of hexadecane increases and so the BPA precipitates, creating nucleation sites for the internal sphere growth. In contrast, when the authors reduced the initial volume fraction of hexadecane to just 30%, no significant differences were observed between the capsules formed with and without BPA. This article demonstrates the importance of considering the effect that changing the composition of a complex cargo could have on the morphology of microcapsules. In other works which exemplify this, Liu et al. found that when forming polymer microcapsules from a Pickering emulsion template, the ratio between block lengths of a block copolymer within the oil core affected the distribution of hydroxyapatite particles at the oil-water interface, which in turn affected the surface morphology of the resulting microparticles (Liu et al., 2011). The microparticles are formed as the solvent evaporates from the core, resulting in shrinkage of the emulsion droplets, and polymer precipitation. When changing the block lengths of poly(L-lactide-co-ε-caprolactone) P(LA/CL) from 50:50 to 75:25, the interaction between the particles and the polymer becomes weaker due to the reduced carboxyl groups per unit mass, resulting in easier detachment of the particles as shrinkage occurs, and leading to a smoother surface as compared to the crinkled surface of the P(LA/CL) 50:50 microparticles. Similarly, Hitchcock et al. found that adsorption of platinum nanoparticles to a polymer-water interface was affected by the oil core contained within the polymer microcapsule (Tasker et al., 2017; Hitchcock et al., 2018). They found that by replacing toluene with hexyl salicylate in the microcapsule core, the nanoparticles adsorbed to the polymer microcapsules in an aggregated, fractal pattern on the polymer surface, with the equivalent of up to eight monolayers forming on one surface as opposed to the dense monolayer coverage observed on the toluene core microcapsules.

## Conclusion

The increasing use of particle-stabilized fluid-fluid interfaces to template the formation of micro/nanostructured capsules that encapsulate an active component has ultimately led to increasing complexity of the components that make up the template emulsion. This is particularly relevant in the field of drug delivery where the drive to incorporate higher concentrations of drug increases the influence of the physicochemical properties of the drug on the emulsion core. The formation and resulting structure of nature's highly defined self-assembling nanocapsules, viruses, is highly sensitive to the composition of the core template and serves as a guiding example to other microcapsule fields. Indeed, computational simulation of assembly has shown that the strength of the interactions between viral subunits and the core, relative to inter-subunit interactions, is a governing principle on assembly around cores that do not match the preferred empty particle geometry (Elrad and Hagan, 2008). Our review of recent literature suggests that minor changes in core composition, particularly toward more complex systems, can play a critical role in the stability of many fluid-fluid interfaces, by altering the interfacial properties of the system, ultimately impacting the final structure of templated capsules. We believe that continued efforts to understand the fundamental forces that drive the stability of fluid-fluid interfaces in complex mixtures will ultimately underpin further advancement in the field.

## Author contributions

AT and FS jointly led the initial literature search and drafting. All authors contributed equally to the final writing of the manuscript.

### Conflict of interest statement

The authors declare that the research was conducted in the absence of any commercial or financial relationships that could be construed as a potential conflict of interest.

## References

[B1] Ali M. McCoy T. M. McKinnon I. R. Majumder M. Tabor R. F. (2017). Synthesis and characterization of graphene oxide–polystyrene composite capsules with aqueous cargo via a water–oil–water multiple emulsion templating route. ACS Appl. Mater. Interfaces 9, 18187–18198. 10.1021/acsami.7b02576 28492312

[B2] Amalvy J. I. Unali G. F. Li Y. Granger-Bevan S. Armes S. P. Binks B. P.. (2004). Synthesis of sterically stabilized polystyrene latex particles using cationic block copolymers and macromonomers and their application as stimulus-responsive particulate emulsifiers for oil-in-water emulsions. Langmuir 20, 4345–4354. 10.1021/la035921c 15969137

[B3] Ashley C. E. Carnes E. C. Phillips G. K. Durfee P. N. Buley M. D. Lino C. A.. (2011). Cell- specific delivery of diverse cargos by bacteriophage MS2 virus-like particles. ACS Nano 5, 5729–5745. 10.1021/nn201397z 21615170 PMC3144304

[B4] Aveyard R. Binks B. P. Clint J. H. (2003). Emulsions stabilised solely by colloidal particles. Adv. Colloid Interfaces Sci. 100–0102, 503–546. 10.1016/S0001-8686(02)00069-6

[B5] Binks B. P. Clint J. H. (2002). Solid wettability from surface energy components: relevance to pickering emulsions. Langmuir 18, 1270–1273. 10.1021/la011420k

[B6] Binks B. P. Dyab A. K. F. Fletcher P. D. I. (2007). Contact angles in relation to emulsions stabilised solely by silica nanoparticles including systems containing room temperature ionic liquids. Phys. Chem. Chem. Phys. 9, 6391–6397. 10.1039/b711174f 18060169

[B7] Brasch M. Putri R. M. De Ruiter M. V. Luque D. Koay M. S. Caston J. R.. (2017). Assembling enzymatic cascade pathways inside virus-based nanocages using dual-tasking nucleic acid tags. J. Am. Chem. Soc. 139, 1512–1519. 10.1021/jacs.6b10948 28055188 PMC5330652

[B8] Chang C. B. Knobler C. M. Gelbart W. M. Mason T. G. (2008). Curvature dependence of viral protein structures on encapsidated nanoemulsion droplets. ACS Nano 2, 281–286. 10.1021/nn700385z 19206628

[B9] Chudasama V. Maruani A. Caddick S. (2016). Recent advances in the construction of antibody-drug conjugates. Nat. Chem. 8, 113–118. 10.1038/nchem.2415 26791893

[B10] Duncan R. (2011). Polymer therapeutics as nanomedicines: new perspectives. Curr. Opin. Biotechnol. 22, 492–501. 10.1016/j.copbio.2011.05.507 21676609

[B11] Elrad O. M. Hagan M. F. (2008). Mechanisms of size control and polymorphism in viral capsid assembly. Nano Lett. 8, 3850–3857. 10.1021/nl802269a 18950240 PMC2742690

[B12] Enomoto T. Kawano M. Fukuda H. Sawada W. Inoue T. Haw K. C.. (2013). Viral protein-coating of magnetic nanoparticles using simian virus 40 VP1. J. Biotechnol. 167, 8–15. 10.1016/j.jbiotec.2013.06.005 23791947

[B13] Frasch-Melnik S. Norton I. T. Spyropoulos F. (2010). Fat-crystal stabilised w/o emulsions for controlled salt release. J. Food Eng. 98, 437–442. 10.1016/j.jfoodeng.2010.01.025

[B14] Ghosh S. Rousseau D. (2011). Fat crystals and water-in-oil emulsion stability. Curr. Opin. Colloid Interfaces Sci. 16, 421–431. 10.1016/j.cocis.2011.06.006

[B15] Glasgow J. E. Capehart S. L. Francis M. B. Tullman-Ercek D. (2012). Osmolyte-mediated encapsulation of proteins inside MS2 viral capsids. ACS Nano 6, 8658–8664. 10.1021/nn302183h 22953696 PMC3479312

[B16] Han F. Y. Thurecht K. J. Whittaker A. K. Smith M. T. (2016). Bioerodable PLGA-based microparticles for producing sustained-release drug formulations and strategies for improving drug loading. Front. Pharmacol. 7:185. 10.3389/fphar.2016.00185 27445821 PMC4923250

[B17] He J. Niu Z. Tangirala R. Wang J.-Y. Wei X. Kaur G.. (2009). Self-assembly of tobacco mosaic virus at oil/water interfaces. Langmuir 25, 4979–4987. 10.1021/la803533n 19397351

[B18] Hitchcock J. P. Tasker A. L. Stark K. Leeson A. Baxter E. A. Biggs S.. (2018). Adsorption of catalytic nanoparticles onto polymer substrates for controlled deposition of microcapsule metal shells. Langmuir 34, 1473–1480. 10.1021/acs.langmuir.7b02874 29227687

[B19] Hu Y. Zandi R. Anavitarte A. Knobler C. M. Gelbart W. M. (2008). Packaging of a polymer by a viral capsid: the interplay between polymer length and capsid size. Biophys. J. 94, 1428–1436. 10.1529/biophysj.107.117473 17981893 PMC2212672

[B20] Kaur G. He J. Xu J. Pingali S. Jutz G. Böker A.. (2009). Interfacial assembly of turnip yellow mosaic virus nanoparticles. Langmuir 25, 5168–5176. 10.1021/la900167s 19354217

[B21] Kawano M. Doi K. Fukuda H. Kita Y. Imai K. Inoue T.. (2015). SV40 VP1 major capsid protein in its self-assembled form allows VP1 pentamers to coat various types of artificial beads *in vitro* regardless of their sizes and shapes. Biotechnol. Rep. 5, 105–111. 10.1016/j.btre.2014.12.008 28435806 PMC5374266

[B22] Kopecek J. (2013). Polymer-drug conjugates: origins, progress to date and future directions. Adv. Drug Deliv. Rev. 65, 49–59. 10.1016/j.addr.2012.10.014 23123294 PMC3565043

[B23] Liu X. Okada M. Maeda H. Fujii S. Furuzono T. (2011). Hydroxyapatite/biodegradable poly(l-lactide–co-ε-caprolactone) composite microparticles as injectable scaffolds by a pickering emulsion route. Acta Biomater. 7, 821–828. 10.1016/j.actbio.2010.08.023 20807593

[B24] Macierzanka A. Szelag H. Szumała P. Pawłowicz R. Mackie A. R. Ridout M. J. (2009). Effect of crystalline emulsifier composition on structural transformations of water-in-oil emulsions: emulsification and quiescent conditions. Colloids Surf. A Physicochem. Eng. Aspects 334, 40–52. 10.1016/j.colsurfa.2008.09.053

[B25] Manuela C. De Souza P. L. Aditya R. Stenzel M. H. (2017). The effect of drug loading on micelle properties: solid-state NMR as a tool to gain structural insight. Angew Chem. 129, 8561–8565. 10.1002/ange.201701471 28570761

[B26] Marchetti M. Wuite G. J. L. Roos W. H. (2016). Atomic force microscopy observation and characterization of single virions and virus-like particles by nano-indentation. Curr. Opin. Virol. 18, 82–88. 10.1016/j.coviro.2016.05.002 27253691

[B27] Mukherjee S. Kler S. Oppenheim A. Zlotnick A. (2010). Uncatalyzed assembly of spherical particles from SV40 VP1 pentamers and linear dsDNA incorporates both low and high cooperativity elements. Virology 397, 199–204. 10.1016/j.virol.2009.10.050 19942248

[B28] Ngai T. Auweter H. Behrens S. H. (2006). Environmental responsiveness of microgel particles and particle-stabilized emulsions. Macromolecules 39, 8171–8177. 10.1021/ma061366k

[B29] Ramazani F. Chen W. L. Van Nostrum C. F. Storm G. Kiessling F. Lammers T.. (2016). Strategies for encapsulation of small hydrophilic and amphiphilic drugs in PLGA microspheres: state-of-the-art and challenges. Int. J. Pharm. 499, 358–367. 10.1016/j.ijpharm.2016.01.020 26795193

[B30] Read E. S. Fujii S. Amalvy J. I. Randall D. P. Armes S. P. (2004). Effect of varying the oil phase on the behavior of pH-responsive latex-based emulsifiers: demulsification vs. transitional phase inversion. Langmuir 20, 7422–7429. 10.1021/la049431b 15323485

[B31] Russell J. T. Lin Y. Böker A. Su L. Carl P. Zettl H.. (2005). Self-assembly and cross-linking of bionanoparticles at liquid–liquid interfaces. Angew Chem. Int. Edn. 44, 2420–2426. 10.1002/anie.200462653 15806611

[B32] Speir J. A. Johnson J. E. (2012). Nucleic acid packaging in viruses. Curr. Opin. Struc. Biol. 22, 65–71. 10.1016/j.sbi.2011.11.002 22277169 PMC3288730

[B33] Tasker A. L. Hitchcock J. Baxter E. A. Cayre D. O. J. Biggs S. (2017). Understanding the mechanisms of gold shell growth onto polymer microcapsules to control shell thickness. Chem. Asian J. 12, 1641–1648. 10.1002/asia.201700536 28544505

[B34] Tasker A. L. Hitchcock J. P. He L. Baxter E. A. Biggs S. Cayre O. J. (2016). The effect of surfactant chain length on the morphology of poly(methyl methacrylate) microcapsules for fragrance oil encapsulation. J. Colloid Interfaces Sci. 484, 10–16. 10.1016/j.jcis.2016.08.058 27572610

[B35] Tasker A. L. Puttick S. Hitchcock J. Cayre O. J. Blakey I. Whittaker A. K. (2018). A two-step synthesis for preparing metal microcapsules with a biodegradable polymer substrate. J. Mater. Chem B 6, 2151–2158. 10.1039/C8TB00348C 32254438

[B36] Vaughan R. Tragesser B. Ni P. Ma X. Dragnea B. Kao C. C. (2014). The tripartite virions of the brome mosaic virus have distinct physical properties that affect the timing of the infection process. J. Virol. 88, 6483–6491. 10.1128/JVI.00377-14 24672042 PMC4093861

[B37] Veverka M. Dubaj T. Veverková E. Šimon P. (2018). Natural oil emulsions stabilized by β-glucan gel. Colloids Surf. A Physicochem. Eng. Aspects 537, 390–398. 10.1016/j.colsurfa.2017.10.043

[B38] Wagdare N. A. Marcelis A. T. M. Boom R. M. Van Rijn C. J. M. (2011). Microcapsules with a pH responsive polymer: influence of the encapsulated oil on the capsule morphology. Colloids Surf. B Biointerfaces 88, 175–180. 10.1016/j.colsurfb.2011.06.028 21764268

[B39] Wang Z. Qiu T. Guo L. Ye J. He L. Li X. (2018). Polymerization induced shaping of Pickering emulsion droplets: from simple hollow microspheres to molecularly imprinted multicore microrattles. Chem. Eng. J. 332, 409–418. 10.1016/j.cej.2017.09.027

[B40] Wen A. M. Steinmetz N. F. (2016). Design of virus-based nanomaterials for medicine, biotechnology, and energy. Chem. Soc. Rev. 45, 4074–4126. 10.1039/C5CS00287G 27152673 PMC5068136

